# Co-registration of eye movements and event-related potentials in connected-text paragraph reading

**DOI:** 10.3389/fnsys.2013.00028

**Published:** 2013-07-10

**Authors:** John M. Henderson, Steven G. Luke, Joseph Schmidt, John E. Richards

**Affiliations:** Department of Psychology, Institute for Mind and Brain, University of South CarolinaColumbia, SC, USA

**Keywords:** eyetracking, event-related potentials (ERPs), reading, eye movements, coregistration, pseudo-reading

## Abstract

Eyetracking during reading has provided a critical source of on-line behavioral data informing basic theory in language processing. Similarly, event-related potentials (ERPs) have provided an important on-line measure of the neural correlates of language processing. Recently there has been strong interest in co-registering eyetracking and ERPs from simultaneous recording to capitalize on the strengths of both techniques, but a challenge has been devising approaches for controlling artifacts produced by eye movements in the EEG waveform. In this paper we describe our approach to correcting for eye movements in EEG and demonstrate its applicability to reading. The method is based on independent components analysis, and uses three criteria for identifying components tied to saccades: (1) component loadings on the surface of the head are consistent with eye movements; (2) source analysis localizes component activity to the eyes, and (3) the temporal activation of the component occurred at the time of the eye movement and differed for right and left eye movements. We demonstrate this method's applicability to reading by comparing ERPs time-locked to fixation onset in two reading conditions. In the text-reading condition, participants read paragraphs of text. In the pseudo-reading control condition, participants moved their eyes through spatially similar pseudo-text that preserved word locations, word shapes, and paragraph spatial structure, but eliminated meaning. The corrected EEG, time-locked to fixation onsets, showed effects of reading condition in early ERP components. The results indicate that co-registration of eyetracking and EEG in connected-text paragraph reading is possible, and has the potential to become an important tool for investigating the cognitive and neural bases of on-line language processing in reading.

## Introduction

Natural reading is a highly skilled activity that draws on most of the major perceptual and cognitive faculties of the human brain, including perception, attention, motor control, language processing, and reasoning. Two important techniques for investigating many of the sub-processes of reading have been eyetracking and event-related potentials (ERPs). A major motivation for the development of eyetracking methods historically (e.g., Dodge, 1901; Huey, 1908; see Rayner and Pollatsek, 2013) was the study of skilled reading.

During reading, the eyes move across the page at a rate of about four fixations per second. Most words in a text are fixated, and many words receive more than one fixation. Shorter, higher frequency, and more highly constrained words tend to be skipped more often than longer, lower frequency, and less constrained words. The majority of saccades carry the eyes forward (rightward in English) through the text, though backward or regressive eye movements are not uncommon. The eyes also move right to left during return sweeps, taking them from the end of one line to the beginning of the next. Fixation durations in reading are about 225 ms on average, and average forward saccade amplitudes are about eight character spaces or two degrees for normal text at a typical reading distance, with considerable variability for both measures. The durations of individual fixations on a word as well as cumulative gaze durations are related to the perceptual and cognitive processes associated with that word. For example, the duration of the first fixation on a word is affected by lexical factors (e.g., word length and word frequency), syntactic factors (e.g., syntactic complexity), and discourse factors (e.g., anaphor resolution). Because of its temporal and spatial sensitivity and the fact that it is an “online” measure in the sense that effects of variables of interest show up very rapidly in the eye movement record (e.g., within the fixation on a word of interest), eyetracking has proved to be one of the richest and most important behavioral sources of information about the perceptual, cognitive, and linguistic processes that take place during reading (for reviews, see Henderson, 2013; Rayner and Pollatsek, 2013).

Despite its strengths as a research method for studying reading, one drawback of eyetracking is that it does not provide a direct measure of neural activity. For this reason, investigators have often turned to ERPs in the study of reading and language processing. The vast majority of this work has involved presenting one word at a time in the center of the display while the participant holds fixation (see Kutas and Van Petten, 1994; Kutas and Federmeier, 2011). However, from the perspective of understanding the underlying cognitive and neural processes involved in reading, we would ideally like to be able to combine the spatial and temporal sensitivity of eyetracking with the temporal and neural sensitivity of ERPs (Sereno et al., 1998; Sereno and Rayner, 2003). Moreover, because skilled reading involves sequential motor activity in a series of eye movements, we would like to be able to combine eyetracking and ERPs in connected-text reading. Finally, we would want to precisely co-register these two measures in time with high temporal resolution so that specific ERP components can be linked to specific eye movement activities (like the beginning of a fixation) to generate fixation-based event related potentials.

Until recently there had been little successful work co-registering eyetracking and ERPs. However, in the last few years several groups of researchers have demonstrated that it is possible to control for the EEG activity generated by eye movements in complex tasks and to produce interpretable ERP waveforms (Marton and Szirtes, 1988a,b; Takeda et al., 2001; Graupner et al., 2007; Hutzler et al., 2007; Jagla et al., 2007; Simola et al., 2009; Ossandon et al., 2010; Rama and Baccino, 2010; Dimigen et al., 2011; Kamienkowski et al., 2012; see also Thickbroom and Mastaglia, 1985; Thickbroom et al., 1991). A relatively small number of these studies have examined ERPs in normal (connected-text) reading (Marton and Szirtes, 1988a,b; Dimigen et al., 2011). This work is therefore in its early days, but the technique has enormous potential for increasing our understanding of basic processes related to reading.

The present paper represents our approach to combining eyetracking and ERP in normal reading. We describe our methods for combining data collection using eyetracking and ERP, for co-registering timing across these two methods, and for removing the eye movement artifacts from the EEG data. Several of these techniques are novel in their application to co-registration of eye movements and EEG. Specifically, we base our procedure on independent components analysis combined with source localization, and use three criteria for identifying components tied to saccades: First, component loadings on the surface of the head have to be consistent with eye movements; second, source analysis has to localize component activity to the eyes, and third, the temporal activation of the component has to occur at the time of the eye movement and differ for right and left eye movements.

In addition to a description of our methods, we report results from a new manipulation that allowed us to determine the degree to which effects observed in connected-text reading are due to higher-level cognitive processes related to reading rather than to lower-level processes related to programming and executing sequences of eye movements. We compared ERPs in paragraph text-reading to ERPs in paragraph pseudo-reading. In the pseudo-reading condition, each letter of the text was replaced by a meaningless geometric shape. This manipulation preserved word length and word spacing as well as overall paragraph spatial structure, but removed all meaning. Several studies in the eye-tracking literature have compared eye movements during reading and pseudo-reading (Vitu et al., 1995; Rayner and Fischer, 1996; Nuthmann et al., 2007; Henderson and Luke, 2012; Luke and Henderson, 2013). Eye movements are quite similar in text-reading and pseudo-reading overall, but with some significant differences, most notably in mean fixation duration. Likewise, ERP studies sometimes compare the electrophysiological responses to words and pseudo-words in order to investigate the time-course of word recognition (e.g., Sereno et al., 1998; Maurer et al., 2005; Segalowitz and Zheng, 2009). For example, Sereno et al. (1998) observed an effect of words versus pseudo-words in early ERP components. The pseudo-words used in these ERP studies are often pronounceable non-words and thus contain more linguistic information than the pseudo-text used in eye-tracking research, but the principle behind both techniques is the same. Thus, our manipulation is common to both eye-tracking and ERP research. This makes the reading vs. pseudo-reading manipulation ideal for testing the effectiveness of the co-registration methods described below.

Nine participants took part in two eye movement conditions. In the text-reading condition, participants read a series of paragraphs presented on a computer screen. In the pseudo-reading condition, participants moved their eyes through text-like stimuli that did not carry any meaning. Previous literature has shown that pseudo-reading in which the letters of text are replaced by a single letter such as Z or by geometric shapes produces basic eye movement behavior that in many ways is similar to eye movement behavior in normal reading (Vitu et al., 1995; Rayner and Fischer, 1996; Nuthmann et al., 2007; Henderson and Luke, 2012; Luke and Henderson, 2013). Therefore, pseudo-reading provides a nice control condition against which to examine influences of text-reading on ERPs. In the reading and pseudo-reading conditions, eye movements and EEG were continuously recorded.

The present study was novel in several important ways. First, unlike previous studies, we presented full paragraphs of text rather than combinations of multiple words (Dimigen et al., 2012) or single lines of text (Dimigen et al., 2011). To our knowledge the present study represents the first report of co-registration of eye movements and ERPs in paragraph reading. Second, we introduced a pseudo-reading condition as a control for text-reading. This study represents the first use of this control condition when combining eyetracking and ERP. Third, we used a novel set of procedures to adjust for the effect of eye movements on the post-fixation EEG waveform, including combining source localization with independent components analysis. Finally, to validate our method, we used a growth-curve analysis to investigate ERP differences across conditions.

## Data collection methods

### Participants

Nine graduate and undergraduate students from the University of South Carolina Community gave informed consent and completed the experiment in accordance with the University of South Carolina Institutional Review Board. They were each paid fifty dollars for participating in the study. All had normal or corrected-to-normal vision by self-report.

### Materials

**Figure 1:**
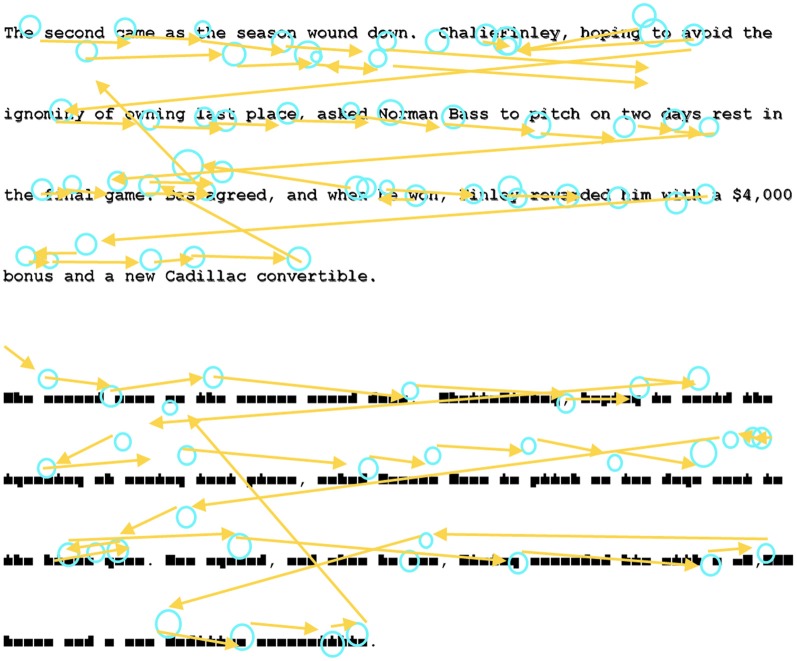
**Example of the Text-Reading and Pseudo-Reading conditions and example scanpaths**.

Fifty-eight short paragraphs (40–60 words) were taken from online news articles. The texts were displayed on the screen in Courier New font. Each paragraph was also converted into pseudo-text using a custom font in which each letter was replaced by a geometric shape that preserved word location and word shapes but eliminated meaning (see Henderson and Luke, 2012; Luke and Henderson, 2013). Both fonts were mono-spaced, and all letters, words, and lines of text appeared in exactly the same location regardless of font. Examples of text-reading and pseudo-reading stimuli can be found in Figure 1.

### Apparatus

Stimuli were presented at a screen resolution of 1024 × 768 pixels using a 28″ LCD monitor. Stimuli were only presented on the middle 2/3 of the screen (approximately 28° × 21°) to accommodate the visual angle of the eye tracker. Eye movements were recorded via an SR Research Eyelink 1000 desktop-mounted eyetracker (spatial resolution of 0.01°) sampling at 1000 Hz. Participants were seated 68 cm from the monitor so that approximately 3.5 characters subtended 1° of visual angle. Head movements were minimized using a chin rest. Viewing was binocular and eye movements were recorded from the right eye. Trials were initiated and terminated with a button box operated by the left hand.

### Procedure

Participants were told that they would be reading short texts on a computer screen while their eye movements were recorded, and that some texts would appear with blocks in place of letters. In the case of the pseudo-texts, participants were instructed to move their eyes “as if they were reading,” consistent with standard pseudo-reading instructions (Vitu et al., 1995; Nuthmann et al., 2007; Henderson and Luke, 2012; Luke and Henderson, 2013). Each paragraph was presented in both the text-reading and pseudo-reading conditions, such that each participant completed 116 trials. The experiment was broken up into four blocks of 29 trials, and the text-reading and pseudo-reading versions of each text were always presented at least one block apart. Within each block, stimuli were presented in a random order for each participant.

#### Eye movement recording

Eyetracking began with a nine-point calibration routine used to map eye position to screen coordinates. Eyetracker calibration was not accepted until the average error was less than 0.49° and the maximum error was less than 0.99°. Participants were recalibrated at the start of each block and as needed during testing. Trials began with participants fixating a point in the upper left corner of the screen and pressing a button on the button box. In addition to initiating the trial, this served as a “drift check” for the eye tracker to record any shift in gaze position since calibration. The fixation point was then replaced by the text, with the first character in the text appearing approximately three degrees below and to the right of the fixation point. The participant was instructed to read through the text and press a button on the button box to end the current trial and proceed to the next trial. One paragraph was presented per trial.

After recording, the eye movement data were analyzed off-line to identify fixations and saccades using the DataViewer software package (SR Research Ltd, version 1.11.1). These data were used in segmenting the EEG data as described below.

#### Recording of EEG and segmenting of EEG for ERP

**Figure 2:**
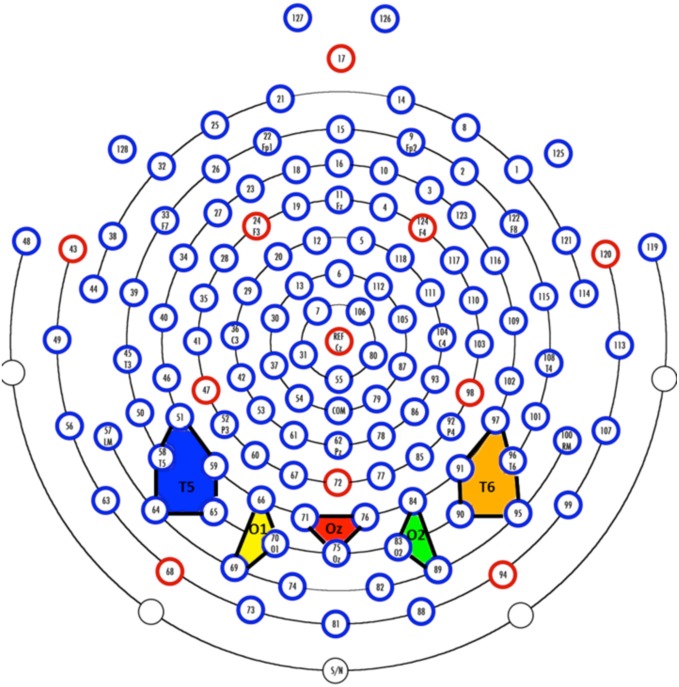
**Virtual electrodes approximating the 10–20 system were created by averaging over groups of electrodes**. The colored polygons show the five most posterior electrode groupings and roughly correspond to T5 (10–20 system; P7 in the 10–10 system; blue), O1 (yellow), Oz (red), O2 (green), and T6 (10–20 system; P8 in the 10–10 system; orange).

The EEG was recorded with a 128 channel system (EGI, Inc., Eugene, OR, USA), referenced to vertex during recording and re-referenced algebraically to an average reference, recorded with 20 K amplification, at a sampling rate of 1 kHz, and with impedances below 100 kΩ. A 128-channel Hydrocel GSN SensorNet (Tucker, 1993; Tucker et al., 1994) was used to record the continuous EEG. The segments for the EEG were initially extracted for the entire session and high-pass filtered with a 0.5 Hz filter. The electrooculogram (EOG) was extracted from the electrodes on the outer canthi (#'s 125 and 128). The saccades in the EOG were identified with a third-order differential filter (Matsuoka and Harato, 1983; Matsuoka and Ueda, 1986). We aligned the saccades found in the EOG with the saccade onsets defined by Dataviewer to ensure consistency between the EEG recording and the eye tracker. The EEG segments were extracted for 100 ms preceding each saccade, through the saccade, and up to 750 ms following the saccade. The segments were terminated if (1) a blink occurred, (2) a return sweep occurred, carrying the eyes from the end of a line to the beginning of the next line, or (3) the recording interval ended. For the ERP analysis, the electrodes were grouped into sets of electrodes from the 128 channel GSN Sensornet into 20 “virtual 10–20” electrodes. The five most posterior electrode groups are shown in Figure 2. Given that we were primarily interested in early ERP components, these five electrodes were the most relevant for analysis.

#### Co-registration of eyetracking and EEG

The EEG recordings and the eyetracking data were aligned so that saccade onset from the eyetracker could be found for each EEG segment. This was accomplished by controlling the experiment via EPrime. During the experiment, the EPrime program had access to the SR-Eyelink time with ms resolution. These times and the EPrime computer time were sent to the EGI system at the beginning of each paragraph by a dedicated TCP/IP port. Events were simultaneously saved in the continuous EEG stream and in the eye movement record. The EGI Netstation recording program kept the trial onset code and exported the trial times with the EEG data. The time streams from the EPrime computer, the EGI computer, and the SR computer could then be integrated into a single time stream for all event codes from the SR eyetracker (saccades, fixations, blinks) and the EEG data.

## Data analysis methods: eye movement correction in EEG data

### Fixation acceptance criteria

Fixations that met the following criteria were included in the analyses. First, fixations had to be preceded by a rightward saccade. Second, fixations could not be followed within 700 ms by a return sweep. Third, fixations could not include a blink. These criteria were identified in the SR eyetracker data using Dataviewer. Fourth, of these fixations, those that were not clearly identifiable in the EOG were excluded. In total this resulted in the inclusion of over 25,000 fixations across all participants; on average each participant contributed approximately 1500 fixations in the text-reading condition and 1300 fixations in the pseudo-reading condition.

### Independent components analysis of ERP data

The complete segmented ERP file was analyzed with Independent Components Analysis (ICA). A spatiotemporal ICA was conducted over the raw EEG data following the procedures outlined by Makeig and colleagues (Makeig et al., 1996, 1997; Jung et al., 2001a,b; DeLorme et al., 2002; also see Richards, 2005; Reynolds and Richards, 2009). The spatiotemporal ICA used the channels as variables, and the observations were all EEG segments from a single participant concatenated over the millisecond intervals for which the EEG was sampled. The weights were calculated using the extended-ICA algorithm of Lee et al. (1999), using sphering of the input matrix to aid in convergence, with an initial learning rate of 0.003. The ICAs were carried out separately on each participant's data.

### Realistic source analysis of eye-movement ICA components using MRI

A structural (anatomical) MRI was taken for each participant. The MRI was used to locate electrodes on the head, develop a realistic finite element method (FEM) head model whose source locations included the eyes, and to estimate the cortical sources of ICA components representing eye movements.

The MRI data were collected at the University of South Carolina McCausland Center for Brain Imaging (USC-MCBI) on a Siemens Medical Systems 3T Trio with an overall duration of about 15 min. A 3D T1-weighted “MPRAGE” RF-spoiled rapid flash scan in the sagittal plane, and a T2/PD-weighted multi-slice axial 2D dual Fast Turbo spin-echo scan in the axial plane was used. The USC-MCBI T1 scans had 1 mm^3^ resolution and sufficient field of view to cover from the top of the head down to the neck.

The EEG recording was done with a Hydrocel GSN (HGSN). Following the recording, the participants were placed inside a “Geodesic Photogrammetry System” (GPS) dome, where images from eleven uniquely angled cameras were acquired, the sum of which provided a complete map of each participant's head. The electrode locations in each participant's headspace were found with a triangulation program for the positions of the electrodes in the photos with the GPS software package (Russell et al., 2005). The electrodes were registered to the structural MRI by identifying a set of fiducial electrodes on the net and on the MRI volume, and using point-set registration to fit the electrodes to the head (Richards et al., 2013).

The electrode locations and structural MRIs were used to develop a realistic head model using finite element method models (Richards, 2013). The heads were segmented into constituent media (gray matter, white matter, skull, skin, nasal cavity, muscle, and eyes) and a source model was constructed that consisted of gray matter and eyes. Three-dimensional tetrahedral wireframes were computed that contained the location of each corner of the tetrahedron and the type of material making up the tetrahedron using the MR Viewer module of the EMSE computer program (Source Signal, Inc.). The electrode locations, source locations, and head model were used with EMSE's Data Analysis (Source Signal, Inc.) to estimate the forward model, inverse model, and current density reconstruction (CDR; Darvas et al., 2001) using sLORETA (Pascual-Marqui et al., 1994; Pascual-Marqui, 2002). The sLORETA current density reconstruction was applied to each of the 128 ICA component loadings.

### Removal of eye movement related EEG activity

**Figure 3:**
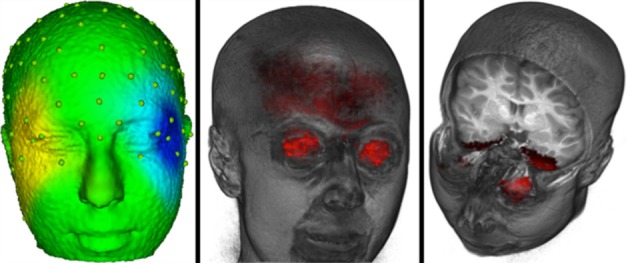
**The eye movement related source localization for one participant**. The left panel shows a scalp potential map with the ICA component loadings of a putative ICA eye movement component for one participant. The center panel shows the eyeball sources. The right panel shows the current density from the cortical source analysis for the ICA component that is shown in the left panel.

Our goal was to analyze the ERP data in the connected paragraph reading conditions with the activity due to eye movements removed from the EEG. To accomplish this, we first removed the electrical activity due to the eye movements from the EEG activity following procedures outlined by Jung et al. (2001a,b). An ICA was performed on all 128 channels using both saccade- and fixation-related ERP segments. Saccade-related segments encompassed 100 ms pre-saccade to 70 ms post-saccade, and fixation-related segments encompassed 20 ms pre-fixation onset to 770 ms post-fixation onset. Note, given that most fixations were less than 770 ms, this resulted in many overlapping segments of data. The ICA component activations were examined at the point of an identified eye movement in the EOG record. Components were identified with activations that occurred primarily around the time of the eye movement. Next we examined the scalp topography of the component loadings. Figure 3 (left panel) shows a scalp potential map with the ICA component loadings of a putative ICA eye movement component. We then used the source analysis based on the realistic head model and the source model (gray matter and eyes) to confirm that the current density in the eyeball location of the source analysis accounted for a substantial portion of the current density across the entire source model. The ratio of the average current density per mm in the eyes, relative to the average current density in the whole head, was computed from the source analysis. We found ratio values greater than approximately 1.5 were sources primarily in the eyeball volume. The right panel of Figure 3 shows the eyeball current density for the same participant shown in the left panel. The eye movement components were identified using three criteria: (1) the component loadings on the surface of the head were consistent with an eye movement, (2) source analysis localized the component to the eyes (ratio of current density in eyes and head >1.5), and (3) the temporal activation of the component occurred at the time of the electrooculogram activity in the eye and differed for right and left eye movements. For the nine participants, the average number of ICA components identified as eye movement components was 9.5 (range = 2 to 21, SD = 6.67). The remaining ICA components were used with loadings/activations to project from the ICA component space back into the temporal EEG space. This resulted in EEG data with the effect of the eye movements removed from the post-fixation EEG data.

**Figure 4:**
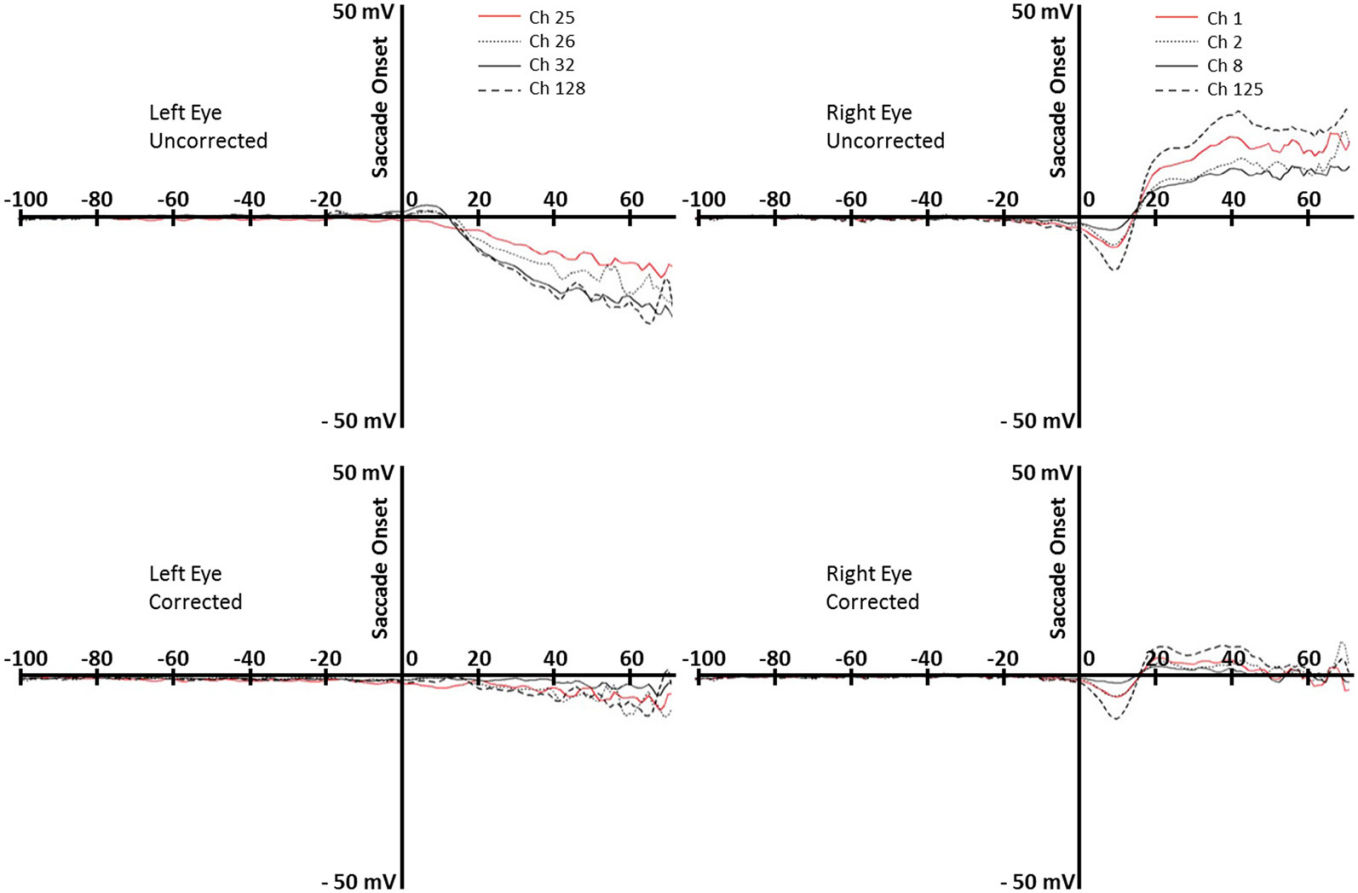
**Top row:** uncorrected data for rightward eye movements during reading, for the left and right eyes (left and right panels). **Bottom row:** corrected data, for the left and right eyes (left and right panels). Data are from the same participant whose data are shown in Figure 3. Each line represents an electrode around the eye (right eye: 1, 2, 8, 125; left eye, 25, 26, 32, 128).

To first assess the goodness of this correction algorithm we examined the saccade locked activity from the channels surrounding the eyes. Figure 4 shows the average EEG recording for the participant shown in Figure 3 from about 100 ms preceding saccades to about 70 ms following saccade onsets for forward-reading eye movements. The top two graphs show the uncorrected EEG recording for the electrodes around the right and left eyes. The EOG-defined saccade onset (sec 0) shows the large EEG changes occurring in these electrodes during the reading eye movement (~30 μV deflection). The bottom two graphs show the corrected EEG recording for the electrodes around the right and left eyes. In the 70 ms after the saccade, there is a small EEG deflection (<10 μV deflection). Note the activity immediately around saccade onset where some electrodes still show a small deflection in the EEG recording.

The eye-movement corrected data were processed as typical EEG data for subsequent analyses. The data were filtered with a 45 Hz low-pass filter and re-referenced to the average of all electrodes. The EEG was segmented at the start of each fixation defined by the timing from the eyetracker through 700 ms after fixation offset. Channels on individual trials were eliminated if there was a voltage change of greater than 50 μV within a segment.

### ERP analysis following eye movement correction

Corrected EEG data were imported into EEGLAB\ ERPLAB for further analysis (version 10.2.5.8b and 3.0.2.1, respectively). Fixation events were matched with the original eye movement data and recoded to include fixation duration and several other eye movement variables. The recoded events were imported using the ERPLAB eventlist function. Across all channels, data from two to six electrodes were averaged together to compute 20 virtual electrodes which approximate standard 10–20 coordinates. Our analyses focused on the five posterior electrodes (see Figure 2). The data were epoched, binned, and averaged by text type and by fixation duration.

## Results

### Eye movement analysis of reading condition

As a manipulation check, we first confirmed an effect of text type in the eye movement data. Replicating previous research (e.g., Vitu et al., 1995; Nuthmann et al., 2007; Henderson and Luke, 2012; Luke and Henderson, 2013), fixation durations were significantly longer in the pseudo-reading (270 ms) than text-reading conditions [220 ms; *F*_(1, 8)_ = 40.1, *MSE* = 373, *p* < 0.001].

### ERP by fixation duration

**Figure 5:**
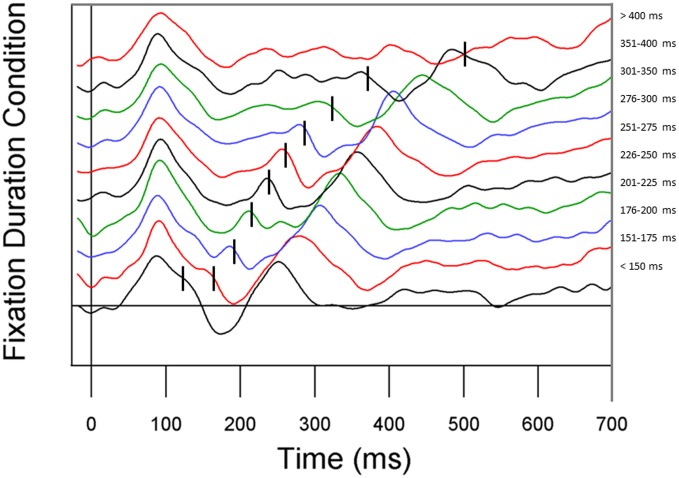
**ERP waveforms stratified by fixation duration**. Fixation duration increases from the bottom line to the top line. From bottom to top, conditions represent all fixations less than 150 ms, 151–175 ms, 176–200 ms, 201–225 ms, 226–250 ms, 251–275 ms, 276–300 ms, 301–350 ms, 351–400 ms, and all fixations greater than 400 ms.

In an initial analysis to determine whether our eye movement correction method was effective, we stratified the EEG data by fixation duration to investigate the effect of eye movements on the fixation-based ERP (see Figure 5). For this analysis, fixation duration bins were chosen to roughly equate for the number of fixations per bin. Replicating Dimigen et al. (2011), we observed clear P1 and N1 components at most fixation durations. However, shortly after fixation offset, the next fixation elicited another P1, overlapping with later ERP components. The fact that P1 components from subsequent fixations appear earlier in the current fixation when the current fixation is shorter can be problematic for interpreting later components in the current fixation.

### Time-course analysis of P1 and N1; growth-curve analysis

We next compared the ERP responses relative to fixation onset in the text-reading and pseudo-reading conditions using Growth Curve Analysis (Mirman et al., 2008) in R (R Development Core Team, 2012). This analysis combines the analyses of amplitude and latency into a single analysis, and treats time as a continuous predictor so that no binning is required. We also performed more traditional analyses of peak amplitude and latency analyses, and the results were highly consistent with the results we report below. The participant-level overall time course for electrodes T5, O1, O2, and T6 was modeled using the ms-by-ms average ERP activity with a third-order (cubic) orthogonal polynomial and a fixed effect of Text Type (Text-Reading vs. Pseudo-Reading) on all time terms.

**Figure 6:**
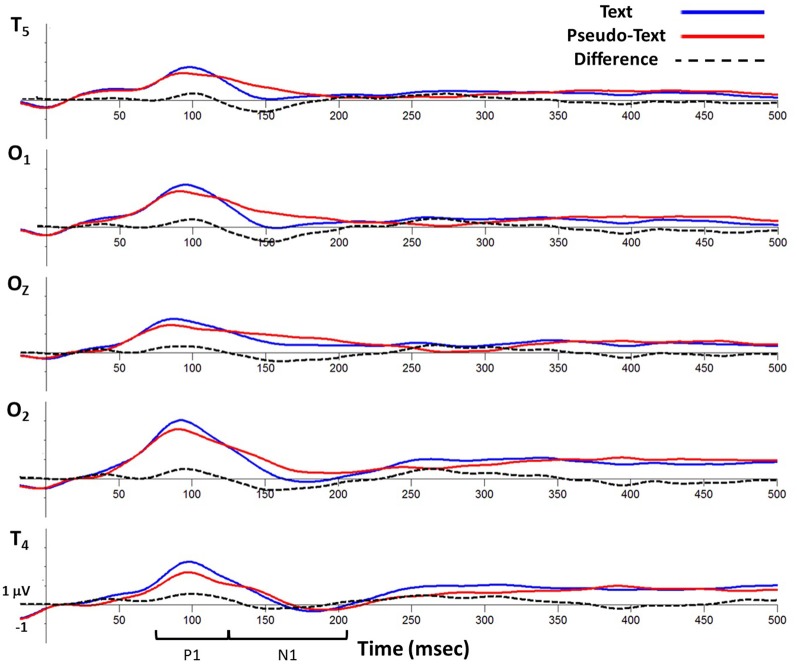
**Grand average waveforms time-locked to fixation onset showing activity at virtual electrode T5 (top), O1, Oz, O2, and T6 (bottom) for the text-reading and pseudo-reading conditions**.

**Figure 7:**
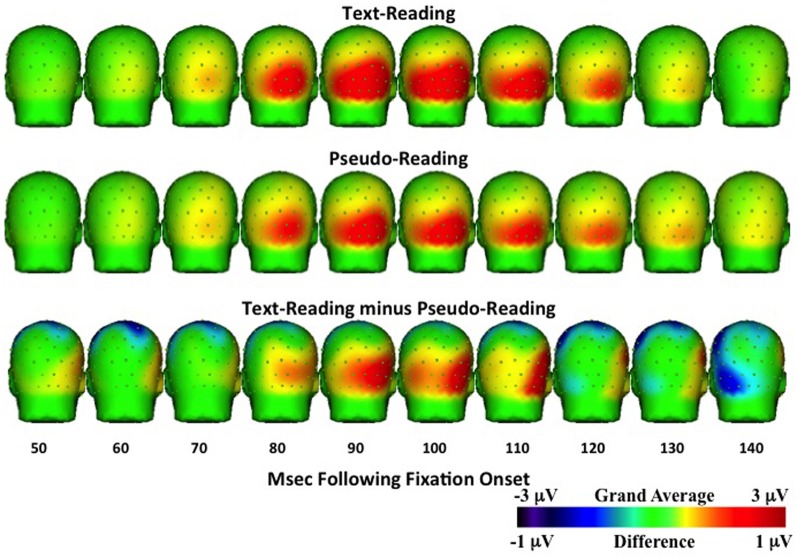
**Temporal topographical scalp potential maps showing grand average ERP activity during the P1 interval (50–150 ms after fixation onset) for text-reading (top row) and pseudo-reading (middle row)**. The bottom row shows the difference wave for this interval.

Analyses of sample data in the P1 time window (75–125 ms) revealed significantly higher overall amplitudes in text-reading than in pseudo-reading at all tested electrodes (all *t*s > 3.65, all *p*s < 0.001; see Figures 6, 7). Additionally, text type interacted with the quadratic term in all analyses (all *t*s > 2.63, all *p*s < 0.05), indicating that the positive wave had a steeper slope in the text condition. The absence of any effect or interaction involving the cubic term indicates similar latencies for text-reading and pseudo-reading. Figure 6 shows the positive activity that occurred during this time window. The positive peak was larger across all tested electrodes for text-reading than for pseudo-reading. The difference wave between text-reading and pseudo-reading ERPs peaked at about 100 ms for all five posterior electrode sites.

**Figure 8:**
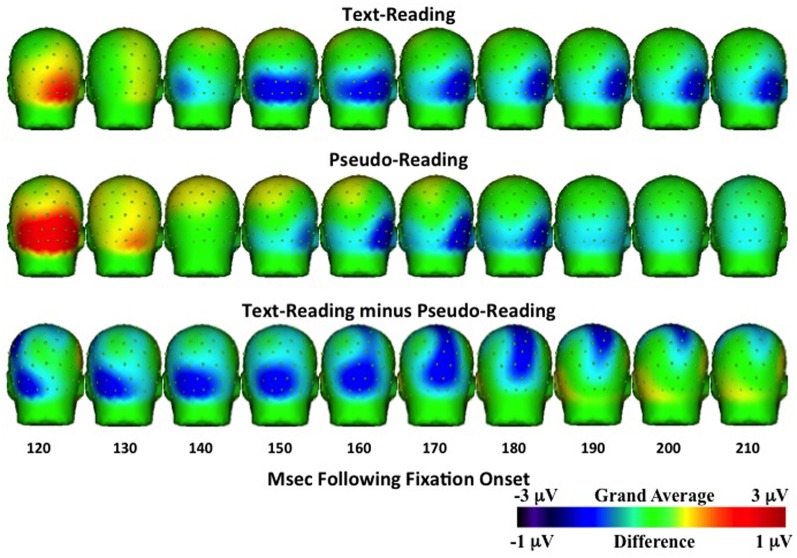
**Temporal topographical scalp potential maps showing grand average ERP activity during the N1 interval (120–220 ms after fixation onset) for text-reading (top row) and pseudo-reading (middle row)**. The bottom row shows the difference wave for this interval. The text- and pseudo-reading maps were created using a 1 Hz high-pass filter to emphasize the negative-going slope of the ERP activity.

For the N1 time window (125–210 ms), amplitudes were significantly more negative for text-reading than pseudo-reading at all tested electrodes (all *t*s > 2.33, all *p*s < 0.05), indicating a larger N1 in that condition (see Figures 6, 8). Text type again interacted with the quadratic term in all analyses (all *t*s > 5.32, all *p*s < 0.001), indicating significantly steeper slopes in text-reading than pseudo-reading. Further, in the analyses of the two left-hemisphere electrodes, the cubic term interacted with text type (both *t*s > 2.33, both *p*s < 0.05) indicating that the cubic was a good fit in the text reading condition but not the pseudo-reading condition. This interaction indicates that the N1 peaked earlier for text-reading than for pseudo-reading at the left hemisphere electrodes.

Figure 6 shows the negative activity occurring in the N1 time window. The ERP during text-reading showed a steeper slope from the peak of the positive component towards the baseline. Further, the difference wave in this figure shows the onset of the difference between text- and pseudo-reading occurred at about 125 ms on the left electrodes and about 150 ms on the right electrodes; and the corresponding peak of the difference wave also occurred earlier for text-reading than for pseudo-reading.

To further investigate the progression of the text-reading versus pseudo-reading effect across the different electrodes, we conducted a similar growth curve analysis on the difference wave (text-reading minus pseudo-reading) in the N1 time window, with electrode as a fixed effect. This analysis compared the curve of the waveform at electrode T5 to the curve at the other electrodes. The cubic polynomial was significant at T5 (coeff. = −0.8, SE = 0.12, *t* = −6.41, *p* < 0.001). The interaction of electrode and the cubic polynomial term was significant for electrodes O2 and T6 (both *t*s > 2.61, both *p*s < 0.05) but not for electrode O1 (*t <* 0.33), indicating that the cubic term fit the data from the two left hemisphere electrodes (T5 and O2) but not the right hemisphere electrodes (O2 and T6). The fact that the cubic term was the best fit on the left and the quadratic on the right over the same time window indicates that the N1 difference wave peaked earlier on the left than on the right. Figure 6 shows the negative activity occurring in the N1 time window. The ERP during text-reading showed a steeper slope from the peak of the positive component towards the baseline. Further, the difference wave in this figure shows the onset of the difference between text- and pseudo-reading occurring at about 125 ms on the left electrodes and about 150 ms on the right electrodes; and the corresponding peak of the difference wave also occurred earlier for text-reading than for pseudo-reading.

Topographical maps of the ERP activity are shown in Figure 7 for the P1 interval and Figure 8 for the N1 interval. These maps illustrate the findings reported above. The positive activity (Figure 7) showed a distribution centered on the scalp ipsilateral to the forward reading eye movement, but with larger peak amplitude for text-reading than pseudo-reading. In contrast to this, the negative activity (Figure 8) showed a faster onset for the text-reading condition than for the pseudo-reading condition, with a “wave” of activity spreading from the left scalp sites toward the right scalp sites. The text condition also had significantly larger amplitude relative to the pseudo-reading condition. Thus, for the activity in the N1 interval, there were differences in the speed, slope, amplitude, and topography of the response. These findings are in line with prior research in which word reading resulted in significantly larger N1 and P1 amplitudes relative to pseudo-word reading (Sereno et al., 1998). This replication suggests that our source-localized ICA eye movement correction of the EEG signal removed the eye-movement-related artifacts in the data well enough to allow the investigation of ERP's time-locked to fixation onset.

## Discussion

Eye movements and ERPs provide two important sources of data for investigating language processing generally and reading in particular. The possibility of co-registering and combining these two measures in connected-text reading has therefore been of wide interest in the psycholinguistics and reading communities, but the technical challenges have been formidable. In the present study our goal was to determine whether meaningful ERPs can be generated from connected-text paragraph reading. We therefore presented a new method for identifying and removing eye movement effects from EEG data, a new control condition for examining language effects in reading, a new paragraph-reading paradigm, and new data regarding early text-based effects in connected reading.

During normal reading, the eyes move through the text in a series of fixations and saccadic eye movements. Because the eye movements themselves generate a great deal of activity in the EEG data, it is difficult to observe subtle effects related to language processing in the ERP waveform. In the present study, we were interested in determining whether we could generate meaningful ERPs in which the event was tied to eye fixations in natural connected-text reading.

To determine whether ERPs related to reading could be distinguished in the resulting ERPs, we also introduced the use of a pseudo-reading control condition. In this pseudo-reading condition, a pseudo-font was used in which letters were replaced by geometric shapes that roughly maintained the look of text while providing no orthographic information. Texts written in this pseudo-font therefore retained letter spacing, word length, word spacing, and overall paragraph spatial structure without providing any meaning. In the experiment, participants were asked to read paragraphs of text and to move their eyes through pseudo-text as though they were reading. Eye movements in these conditions were remarkably similar (though there were also some important subtle differences), so the pseudo-reading condition provides a useful control for eye movements without language processing.

Comparison of the ERPs from the text-reading and pseudo-reading conditions revealed several clear differences in the shapes of the early ERP P1 and N1 components. For example, N1 amplitudes were more negative for text-reading than pseudo-reading at all tested electrodes. The N1 also peaked earlier for text-reading than pseudo-reading. Furthermore, in a comparison of text-reading and pseudo-reading in posterior regions, the difference wave peaked earlier in the left scalp regions than in the right. The N1 has been associated with discrimination processes at fixation (Luck and Vogel, 2000) and with word recognition effects (Sereno et al., 1998; Maurer et al., 2005). For example, Sereno et al. (1998) found differences in N1 for words, pseudo-words, and consonant strings. The differences we observed for text-reading versus pseudo-reading in the present study are consistent with and extend these previous findings to continuous reading, and provide initial validation for the correction methods we have employed. These initial results suggest that it will be worthwhile to refine these methods and pursue them in future studies designed to use co-registered eyetracking and ERPs, particularly with respect to early ERP components.

Eye movement corrections algorithms have been used previously, even in free reading tasks (e.g., Dimigen et al., 2011). The procedure used in the current study compares favorably with those on two points. First, the eye movement correction algorithm introduced here was applied to the actual reading data rather than data from a separate eye movement calibration recording as in other studies (e.g., Dimigen et al., 2011; Plochl et al., 2012). It is likely that the types of eye movements made in calibration trials will be dissimilar to the ocular characteristics of eye movements occurring during reading (e.g., moving eyes at 15° in four cardinal directions, Dimigen et al., 2011; eye movements and blinks on a gray screen, Plochl et al., 2012). Separate eye movement calibration trials could result in models of the eye movement effect on EEG that are dissimilar to what happens during reading. Our *in situ* correction specifically models the eye movements occurring during reading for their effect on the EEG. Our strategy based on individual participants should account for individual differences in reading experience or ability. Second, several ocular correction techniques use tools that do not specifically identify the temporal location of a saccadic eye movement occurring during reading. Most techniques attempt to control for blinks, vertical saccades, small eye movements, and horizontal saccades. However, since we know the onset of the saccade that moves the eye from one word to another from both the eye tracker and the EOG, our method limits the correction to the eye movement activity in the EEG specifically related to the saccade that begins the fixation of interest. The use of the temporal sequence of the ICA activation as a criterion for identifying eye movement ICA components limits our correction to eye movements occurring during reading and excludes other eye movements (blinks, vertical saccades) from our correction. In this respect our method compares favorably to that used by Plochl et al. (2012) which used both ICA temporal activation and component loadings during eye-tracking defined saccadic activity (though see the first point above).

We believe our method is superior to other methods in distinguishing ocular-related EEG activity and neural-related EEG activity caused by eye movements. First, our method identifies the eye-movement related activity in the EEG that is related specifically to the movement of the eyes in the orbit, and does not correct all eye movement related EEG activity. Eye movements may affect EEG activity for “ocular” reasons or “neural” reasons. The ocular effects on EEG occur because of electrical activity generated by the eye. This includes muscle activity around the eyes during eye movements, rotation of the corneal dipole, and muscle/eyelid activity occurring during blinks; often these are labeled “eye movement artifacts” and are the target for correction. However, there is also eye movement-related EEG activity from neural sources. For example, the fixation-related-potentials in this and similar studies are due to neural activity temporally linked to the eye movement, but presumably caused by neural processes related to reading. Some presaccadic ERP's (e.g., presaccadic parietal slow wave, presaccadic spike potential; Richards, 2005, 2013) are neural in origin and likely involved in the neural control of eye movements. Using source analysis to identify ICA components whose primary sources are in the eyes distinguishes ocular-generated EEG potential changes and neurally-generated EEG potential changes. An interesting comparison may be made with the Dimigen et al. (2011) study, which used a technique that forms a source dipole model of eye movements based on a calibration trial, and also did not remove the parietal presaccadic spike potential. A small ERP potential also occurred around fixation onset in our study lateralized on the side of the eye movement (Figure 4, bottom right panel). This is likely the presaccadic spike potential, which is slightly lateralized over the eye electrodes ipsilateral to the eye movements (Richards, 2013). Unlike both the Dimigen et al. (2011) and the Plochl et al. (2012) correction methods, our method specifically corrects for eye movement activity in the EEG of ocular origin and does not remove eye movement related EEG activity of neural origin. Finally, the use of the temporal activation of the ICA alone is insufficient to identify these eye movement components. The additional criteria of the scalp topography of the component loadings being consistent with an eye movement (e.g., Plochl et al., 2012), and of the source of the EEG activity being localized in the eyes, ensures that the spatial source of the corrected activity is due to ocular activity. It is possible that component loading topography and component activations could be sufficient (e.g., Plochl et al., 2012), or that the ocular source analysis and the component activity could be sufficient. The ocular source analysis loosely reflects a particular topographical mapping on the head consistent with the eye movement in the EEG recording, so that using topographical mapping and the component activation would loosely match our three-pronged criteria.

There are a number of important remaining issues that will have to be resolved before the use of ERPs in connected-text reading can reach its full potential, especially with respect to later ERP components. One challenge is related to the fact that fixations have a relatively short duration in reading (e.g., averaging 220 ms for text-reading in the present study). Because of this, ERP effects arising from the fixation after a critical fixation (critical + 1 fixation) will often overlap with the later components from a critical fixation. This issue is illustrated by Figure 5 (see also Dimigen et al., 2011, Figure 2b) where the components from the next fixation overlap earlier in time as the duration of the current fixation decreases. Adding to the challenge, language manipulations that are expected to affect late ERP components associated with fixation on a word in text (e.g., N400) are also likely to affect the durations of those fixations. Therefore, ERP components tied to critical fixation events in connected-text reading may be particularly susceptible to overlap of late components generated by the critical fixation and early components generated by the fixation following the critical fixation. And the nature of this overlap will change as the duration of the critical fixation changes (Figure 5). Dealing with this issue is not simply a matter of removing EEG effects from the eye movements themselves, but also of de-convolving overlapping waveforms containing the information that is of interest. This issue makes using the later components of ERPs in connected reading particularly challenging. However, we are optimistic that further work in this area will help to fulfill the promise of this method.

In this study we focused on using the eye movement data to identify the events of interest for ERPs (fixations) and to provide information (saccade onsets) useful for removing the eye movement effects in the EEG. However, the longer-term promise of the co-registration of eye movements and ERPs is to combine the evidence generated simultaneously from both measures. For example, in studies of reading we are typically interested in how a theoretically motivated manipulation affects measures of language processing. Data relevant to assessing that manipulation often derive from both eyetracking and ERPs, but traditionally these data are generated from separate experiments (e.g., Sereno et al., 1998; Dambacher et al., 2006; Dambacher and Kliegl, 2007). It would be far more powerful to be able to provide evidence from both methods when the data has been collected simultaneously, so that data drawn from each method can then be used to constrain inferences drawn from the other. The present method development is ultimately directed toward achieving this goal.

### Conflict of interest statement

The authors declare that the research was conducted in the absence of any commercial or financial relationships that could be construed as a potential conflict of interest.
